# Heterogeneous Myeloid Cells in Tumors

**DOI:** 10.3390/cancers13153772

**Published:** 2021-07-27

**Authors:** Aixia Dou, Jing Fang

**Affiliations:** Department of Drug Discovery and Biomedical Sciences, University of South Carolina College of Pharmacy, Columbia, SC 29208, USA; douaixia@gmail.com

**Keywords:** monocytes, tumor-associated macrophages, dendritic cells, cancer-related circulating neutrophils, tumor-associated neutrophils, myeloid-derived suppressor cells, tumors, tumor microenvironment, angiogenesis, immunomodulation, chemotherapy, immunotherapy

## Abstract

**Simple Summary:**

It is well known that lymphocytes play a key role in the immunosurveillance for tumors. Accumulating evidence indicates that myeloid cells also have a large impact on tumor development. The tumor-associated myeloid cells (TAMCs) are heterogeneous and exert distinct and even opposing effects on tumor cells and tumor microenvironment (TME). In addition, myeloid cells play a critical role in modulating the behavior of lymphocytes, resulting in immunostimulatory or immunosuppressive TME cues that stimulate or suppress tumor development. Based on the function of myeloid cells in tumors, there are pro-tumor and anti-tumor myeloid cells. They are involved in pleiotropic processes, including growth, survival, differentiation, stemness, invasiveness, dissemination and metastasis of tumor cells, angiogenesis, remodeling of TME, immunomodulation, and response to cancer therapy. Understanding the function and mechanism of TAMCs in tumors will shed light on uncovering novel therapy.

**Abstract:**

Accumulating studies highlight a critical role of myeloid cells in cancer biology and therapy. The myeloid cells constitute the major components of tumor microenvironment (TME). The most studied tumor-associated myeloid cells (TAMCs) include monocytes, tumor-associated macrophages (TAMs), dendritic cells (DCs), cancer-related circulating neutrophils, tumor-associated neutrophils (TANs), and myeloid-derived suppressor cells (MDSCs). These heterogenous myeloid cells perform pro-tumor or anti-tumor function, exerting complex and even opposing effects on all stages of tumor development, such as malignant clonal evolution, growth, survival, invasiveness, dissemination and metastasis of tumor cells. TAMCs also reshape TME and tumor vasculature to favor tumor development. The main function of these myeloid cells is to modulate the behavior of lymphocytes, forming immunostimulatory or immunosuppressive TME cues. In addition, TAMCs play a critical role in modulating the response to cancer therapy. Targeting TAMCs is vigorously tested as monotherapy or in combination with chemotherapy or immunotherapy. This review briefly introduces the TAMC subpopulations and their function in tumor cells, TME, angiogenesis, immunomodulation, and cancer therapy.

## 1. Introduction

Lymphocytes and myeloid cells belong to the adaptive and innate immunity, respectively, defending microbial infection. Mounting evidence suggest that the immune system also plays an essential role at all stages of the development and metastasis of tumors [1,2]. Lymphocytes have been known as the major immunosurveillance for cancer cells. Myeloid cells are also implicated in cancer biology, exerting both pro-tumor and anti-tumor effects. These tumor-associated myeloid cells (TAMCs) regulate the proliferation, survival, stemness, invasiveness and dissemination of tumor cells, promoting or inhibiting tumor development, and metastasis [3,4]. These myeloid cells also regulate tumor progression and metastasis through reshaping tumor microenvironment (TME) and modulating vasculogenesis and angiogenesis [5,6,7,8]. In addition, TAMCs exert pleiotropic effects on lymphocytes, including recruitment of lymphocytes to TME, enhancement or suppression of the proliferation, differentiation, maturation, activation, and function of lymphocytes, leading to immune response, immune anergy, immune tolerance or immunosuppression [9,10,11,12]. Myeloid cells also largely impact on the response to cancer therapy [13,14]. TAMCs are a heterogenous group of mature and immature myeloid cells and the most studied populations include monocytes, tumor-associated macrophages (TAMs), dendritic cells (DCs), cancer-related circulating neutrophils, tumor-associated neutrophils (TANs), and myeloid-derived suppressor cells (MDSCs). These myeloid cells circulate peripheral blood or reside in tissues and are recruited to TME in response to cytokines, chemokines, or mediators secreted by tumor cells, immune cells, or stromal cells in TME [15]. Each of the myeloid cell population is also heterogenous and consists of distinct subsets involved in pro-inflammatory or anti-inflammatory response to microbial infections. Different subsets of myeloid cells also perform pro-tumor or anti-tumor function during tumor development and metastasis. Factors such as cytokines, chemokines, and metabolites in TME also regulate the proliferation, survival, differentiation, and maturation of circulating and infiltrated myeloid cells and reprogram them towards immunosuppressive and pro-tumor properties [16,17]. We aim to summarize the main subpopulations of TAMCs and their physiological functions and how they regulate tumor development and metastasis and response to cancer therapy. 

## 2. Monocytes

### 2.1. Subsets of Monocytes in Physiological Processes

Monocytes are a heterogeneous group of mononuclear phagocytes that circulate peripheral blood and function as innate immunity during inflammation. The majority of circulating monocytes are classical monocytes that express CD14^+^CD16^−^ in humans and Ly6C^high^ in mice [18]. After differentiation from the lineage-committed progenitor cells, the common monocyte progenitor (cMoP) [19,20], classical monocytes exit the bone marrow, following the gradients of chemokines, such as C-C motif chemokine ligand 2 (CCL2) and C-C motif chemokine ligand 7 (CCL7), through expressing chemokine receptors, including C-C motif chemokine receptor 2 (CCR2) [21,22]. During homeostasis, circulating classical monocytes convert to intermediate monocytes that express CD14^+^CD16^+^ in humans and Ly6C^int^ in mice, and subsequently convert to nonclassical monocytes that express CD14^low^CD16^+^ in humans and Ly6C^low^ in mice [23,24]. Upon infection, classical monocytes rapidly extravasate to inflamed tissues in response to chemokines, cytokines, and complement fragments; and mediate antimicrobial effects, such as phagocytosis [21,25].

### 2.2. Function of Monocytes in Tumors

**Figure 1:**
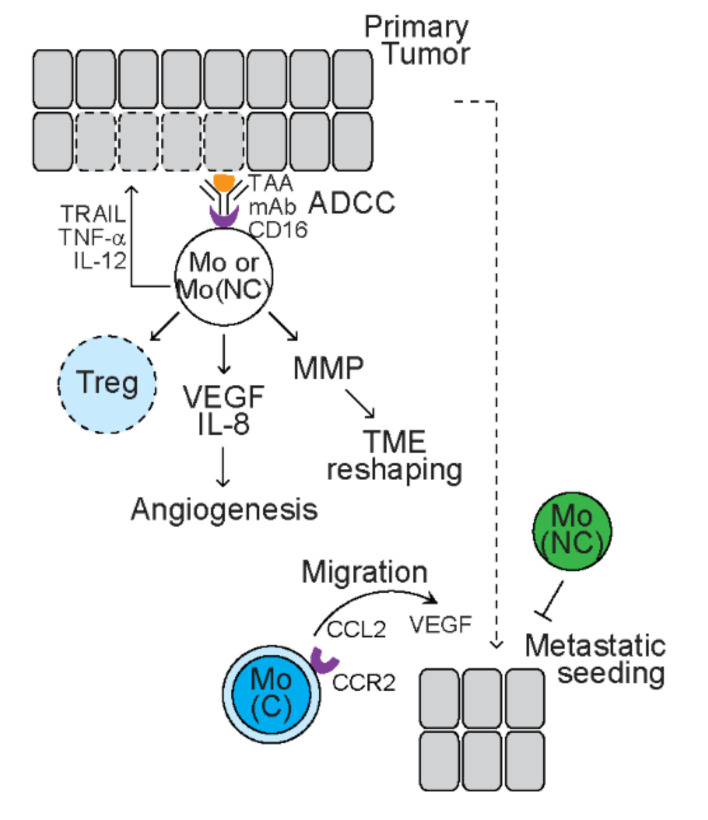
The function of monocytes in tumors. Light blue circles represent human immune cells with pro-tumor function. Dark green circles represent mouse immune cells with anti-tumor function. Dark blue circles represent mouse immune cells with pro-tumor function. White circles represent human immune cells with both anti-tumor and pro-tumor function. Cells with dashed lines represent dead cells. Monocytes (Mo) mediate cytotoxicity of tumor cells through secreting tumor necrosis factor-related apoptosis-inducing ligand (TRAIL), tumor necrosis factor alpha (TNF-α), and interleukin 12 (IL-12) and antibody-dependent cellular cytotoxicity (ADCC). Monocytes promote angiogenesis through upregulating vascular endothelial growth factor (VEGF) and interleukin 8 (IL-8). Monocytes reshape tumor microenvironment (TME) through producing matrix metalloproteinases (MMP). Nonclassical monocytes (Mo(NC)) mediate cytotoxicity of regulatory T cells (Tregs). C-C motif chemokine receptor (CCR2)^+^ classical monocytes (Mo(C)) are recruited by C-C motif chemokine ligand 2 (CCL2) and promote metastatic seeding by secreting VEGF, while Mo(NC) reduce metastasis. TAA: tumor-associated antigen; mAb: monoclonal antibodies.

At different stages of tumor development, different subsets of monocytes exhibit diverse and even opposing effects (Figure 1). Treatment with interferon gamma (IFN-γ) or interferon alpha (IFN-α) upregulates the expression of tumor necrosis factor-related apoptosis-inducing ligand (TRAIL) but downregulates the expression of TRAIL receptor 2 in human monocytes [26]. TRAIL mediates apoptosis of cancer cells without injuring monocytes [26]. Following stimulation with tumor cells, human CD14^+^CD16^+^ monocytes increase the production of pro-inflammatory cytokines tumor necrosis factor alpha (TNF-α) and interleukin 12 (IL-12) but reduce the production of anti-inflammatory cytokine interleukin 10 (IL-10), and exert direct cytotoxicity of tumor cells [27]. In the presence of anti-tumor monoclonal antibody (mAb), human monocytes cultured with macrophage colony-stimulating factor (M-CSF, also referred as CSF-1) phagocytose melanoma and neuroblastoma tumor cells, a process known as ‘antibody-dependent cellular cytotoxicity’ (ADCC) [28]. CD16, also known as FcγRIII, is the receptor of Fc domain of immunoglobulin G (IgG). Engagement of CD16 in response to mAb-coated tumor cells induces the secretion of TNF-α that mediate cell death of tumor cells expressing TNF-α receptor [29]. These studies reveal that monocytes exhibit tumoricidal activity.

In addition, nonclassical monocytes mediate cytotoxicity of regulatory T cells (Tregs), a group of CD4^+^ helper T (Th) cells with immunosuppressive function, in vitro in an Fcγ-dependent manner [30]. Circulating monocytes from patients and mice with renal cell carcinoma (RCC) display a pro-tumor gene signature with upregulated expression of pro-angiogenic factors *interleukin 8* (*IL-8*) and *vascular endothelial growth factor (VEGF)*, as well as matrix metalloproteinases (MPPs), including *MMP19* and *MMP10* [31]. Functional studies reveal that media from RCC monocytes facilitate angiogenesis that is reversed by blocking VEGF receptor 2 (VEGFR2), indicating that RCC monocytes exert a VEGF-dependent pro-angiogenic property [31]. In addition, media from RCC monocytes facilitate invasiveness of RCC cells that is reversed by MMP inhibitors, indicating that RCC monocytes promote tumor cell invasion through reshaping TME.

In syngeneic mice bearing mammary tumors with spontaneous pulmonary metastases, Gr1^+^Ly6C^+^ mouse classical monocytes are recruited to the pulmonary metastases, which is dependent on CCL2/CCR2 signaling [15]. In nude mice bearing metastatic breast cancer cells, CD14^+^CD16^−^ human classic monocytes are also preferentially recruited to the lungs [15]. Blockade of the CCL2/CCR2 signaling axis inhibits the recruitment of mouse and human classical monocytes to the lungs, leading to less metastasis and prolonged survival time of tumor-bearing mice [15]. Mechanistically, classical monocytes produce VEGF that promotes extravasation of tumor cells, leading to metastatic seeding [32]. In comparison, nonclassical monocytes are activated after engulfing tumor cell-derived microparticles, leading to reduced metastasis of tumor cells to lungs [33]. These studies suggest that classical monocytes exert pro-metastatic effects, while nonclassical monocytes exert anti-metastatic effects.

## 3. Tumor-Associated Macrophages (TAMs)

### 3.1. Subpopulations of Macrophages in Physiological Processes

**Figure 2:**
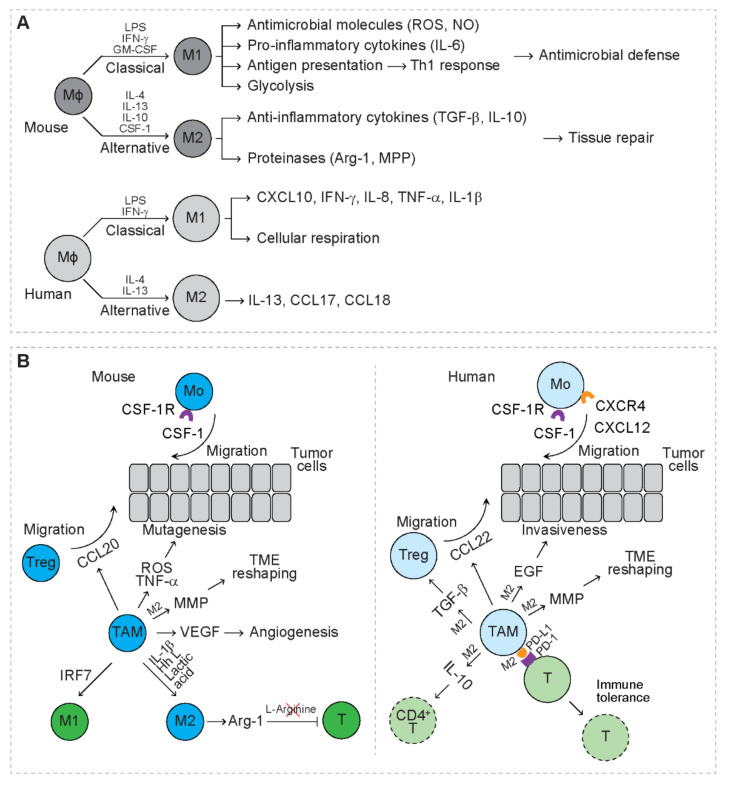
The function of TAMs in tumors. (**A**) Subpopulations of macrophages in physiological processes. Dark grey circles represent mouse macrophages. Light grey circles represent human macrophages. After classical activation, macrophages (Mϕ) are polarized to M1 macrophages (M1); after alternative activation, macrophages are polarized to M2 macrophages (M2). LPS: lipopolysaccharide; IFN-γ: interferon gamma; GM-CSF: granulocyte-macrophage colony-stimulating factor; ROS: reactive oxygen species; NO: nitric oxide; IL-6: interleukin 6; IL-4: interleukin 4; IL-13: interleukin 13; IL-10: interleukin 10; CSF-1: macrophage colony-stimulating factor; TGF-β: transforming growth factor beta; Arg-1: arginase-1; MMP: matrix metalloproteinases; CXCL10: C-X-C motif chemokine 10; IL-8: interleukin 8; TNF-α: tumor necrosis factor alpha; IL-1β: interleukin 1 beta; CCL17/18: C-C motif chemokine ligand 17/18. (**B**) Mouse (left) and human (right) macrophages in tumors. Dark green circles represent mouse immune cells with anti-tumor function. Dark blue circles represent mouse immune cells with pro-tumor function. Light green circles represent human immune cells with anti-tumor function. Light blue circles represent human immune cells with pro-tumor function. Cells with dashed lines represent dead cells. Macrophage colony-stimulating factor 1 (CSF-1)/macrophage colony-stimulating factor 1 receptor (CSF-1R) axis mediates recruitment of mouse and human monocytes (Mo) to tumors. C-X-C chemokine ligand 12 (CXCL12)/ C-X-C motif chemokine receptor 4 (CXCR4) axis mediates recruitment of human monocytes (Mo) to tumors. Mouse tumor-associated macrophages (TAM) are polarized to M1 or M2 macrophages depending on TME cues. M2 TAMs inhibit T cell function through upregulating programmed death-ligand 1 (PD-L1) and secreting interleukin (IL-10). TAMs secrete C-C motif chemokine ligand 20 (CCL20) in mice and C-C motif chemokine ligand 22 (CCL22) in humans to recruit regulatory T cells (Treg). Human TAMs secrete TGF-β to enhance Treg function. M2 TAMs express Arg-1 to inhibit T cell proliferation by depleting L-Arginine. Human TAMs secrete epidermal growth factor (EGF) to enhance invasiveness of tumor cells. Mouse and human TAMs secrete MMP to reshape tumor microenvironment (TME). Mouse TAMs secrete vascular endothelial growth factor (VEGF) to induce angiogenesis.

Tissue resident macrophages serve as the mononuclear phagocyte system, playing a critical role in tissue homeostasis and inflammation [34]. Mouse macrophages are derived from embryonic precursors during embryogenesis and locally self-renew or from circulating monocytes that are released from the bone marrow [35,36]. Macrophages are functionally heterogeneous and divided into two main subpopulations, M1 and M2 macrophages (Figure 2A) [37,38,39]. In response to lipopolysaccharide (LPS), IFN-γ and granulocyte-macrophage colony stimulating factor (GM-CSF), M1 macrophages undergo classical activation and preferentially secrete antimicrobial molecules and pro-inflammatory cytokines, including reactive oxygen species (ROS), nitric oxide (NO), and interleukin 6 (IL-6) [39,40]. M1 macrophages function as the first line defense to fight microbial infections [39,40,41]. M1 macrophages also maintain strong antigen presenting capacity that induces strong Th1 response [39,40,41]. In response to interleukin 4 (IL-4), interleukin 13 (IL-13), IL-10, and CSF-1, M2 macrophages undergo alternative activation and preferentially secrete anti-inflammatory cytokines, including transforming growth factor beta (TGF-β) and IL-10 as well as proteinases (i.e., arginase-1 and MPPs) [40,42]. M2 macrophages play a key role in limiting immune responses, while inducing angiogenesis and tissue repair [43]. Compared to mouse macrophages, human macrophages are less studied. In the presence of LPS and IFN-γ, circulating human macrophages are polarized to M1, secreting C-X-C motif chemokine ligand 10 (CXCL10), IFN-γ, IL-8, TNF-α, and interleukin 1 beta (IL-1β [44]. In the presence of IL-4 and IL-13, human macrophages are polarized to M2, secreting IL-13, C-C motif chemokine ligand 17 (CCL17) and C-C motif chemokine ligand 18 (CCL18) [44]. In response to LPS, mouse macrophages mainly depend on glycolysis for energy, while human macrophages mainly rely on cellular respiration [45]. In response to IL-4, human and mouse macrophages exhibit overlapping and distinct gene signatures [46]. These indicate that human and mouse macrophages perform conserved as well as distinct functions, which needs further investigation.

### 3.2. Recruitment and Polarization of TAMs in TME

In patients with triple-negative breast cancer, the accumulation of CD68^+^ infiltrating macrophages is correlated with a higher risk of metastasis and shortened survival time [47], indicating that TAMs serve as a marker of unfavorable prognosis. The number of CD163^+^ M2-like macrophages increases in bone marrow of patients with acute myeloid leukemia (AML) or multiple myeloma (MM) [48,49]. In patients with T-cell leukemia/lymphoma, the accumulation of CD68^+^CD163^+^ M2-like macrophages is associated with worse clinical outcome [50]. In leukemia mouse models driven by expression of leukemic oncogenes, such as *MLL-AF9*, *AML1-ETO9a*, or *NUP98-HOXD13*, infiltration of macrophages, identified as CD11b^high^Gr1^int^ or CD11b^+^Ly6G^−^ cells, in bone marrow and spleens inversely correlates with the survival of mice [48]. In contrast, in patients with non-small cell lung cancer (NSCLC), the density of CD68^+^HLA-DR^+^ M1-like macrophages in tumor islets is associated with extended survival time [51]. Therefore, the presence of M2-like TAMs is associated with pro-tumor activity, while the presence of M1-like TAMs is associated with anti-tumor activity.

Interaction of myeloma cells and bone marrow stromal cells (BMSCs) upregulates the production of C-X-C motif chemokine ligand 12 (CXCL12), leading to increased recruitment of human monocytes expressing C-X-C motif chemokine receptor 4 (CXCR4) to myeloma TME (Figure 2B) [49]. Blockage of CXCR4 largely inhibits the recruitment of monocytes to myeloma-derived medium [49], indicating that circulating monocytes serve as the main source of TAMs. In patients with breast cancer, upregulation of CSF-1 and CSF-1 receptor (CSF-1R) is associated with inferior prognosis [52]. In a mammary cancer mouse model, CSF-1 promotes the growth of breast cancer cells and metastatic potential through recruiting TAMs to TME [52]. In a pancreatic ductal adenocarcinoma mouse model, both circulating Ly6C^high^ classical monocytes and tissue-resident macrophages contribute to the accumulation of TAMs in TME [53]. Once recruited to TME, TAMs are educated by specific TME cues, such as the molecules derived from tumor cells, stromal cells or immune cells (Figure 2B) [54,55]. In RCC-bearing mice, IL-1β secreted in TME reprograms TAMs towards an M2-like phenotype by downregulating the expression of interleukin 12B (IL-12B) and nitric oxide synthase 2 (NOS2) and upregulating the expression of IL-10 and arginase-1 [31]. With an immunocompetent mouse model, breast cancer cells drive M2 polarization of TAMs through secreting Hedgehog (Hh) ligand [56]. Lactic acid, the end product of glycolysis, is produced by tumor cells and is shown to drive M2 polarization of TAMs via activation of the hypoxia inducible factor 1 subunit alpha (HIF-1α) signaling [17]. In leukemia mouse models, interferon regulatory factor 7 (IRF7) induces M1 polarization of TAMs, which is associated with prolonged survival of leukemia mice [57]. 

### 3.3. Function of TAMs in Tumors

TAMs create a mutagenic microenvironment that favors tumor initiation through secreting pro-inflammatory mediators, such as TNF-α and ROS (Figure 2B) [3,58]. In an intestinal cancer model, H_2_O_2_ produced by myeloid cells triggers DNA mutations, resulting in invasive growth of cancer cells [3]. 

In patients with NSCLC, expression of the immune checkpoint ligand, programmed cell death 1 ligand 1 (PD-L1), is upregulated in tumor-infiltrating immune cells (IC) that are enriched for M2-like TAMs [59]. NSCLC patients with PD-L1^+^ IC exhibit significantly lower disease-free survival rate and overall survival rate than those with PD-L1^−^ IC [59]. In patients with cholangiocarcinoma (CCA), TAMs are positive for PD-L1 and PD-L1^+^ TAMs facilitate CCA progression in mouse model [60]. However, in patients with primary testicular lymphoma, the accumulation of PD-L1^+^ TAMs correlates with favorable survival [61]. Mechanistic studies reveal that expression of PD-L1 is upregulated on TAMs after exposure to lactic acid [62]. Interaction of PD-L1 with programmed cell death 1 (PD-1) on T cells inhibits T cell proliferation and induces T cell apoptosis, leading to immune tolerance (Figure 2B) [62]. TAMs from breast cancer patients suppress the expansion of CD4^+^ helper T cells by expressing PD-L1 and secreting the anti-inflammatory cytokine IL-10 [63]. TAMs of ovarian carcinoma patients secrete C-C motif chemokine ligand 22 (CCL22) to recruit Tregs, which is associated with reduced survival rate of the patients [64]. In mice bearing colorectal cancer, TAMs secrete C-C motif chemokine ligand 20 (CCL20) to recruit Tregs that promote the development of cancer [65]. In addition, human TAMs also secrete TGF-β to enhance the function of Tregs through activation of the forkhead box p3 (Foxp3) signaling pathway [66]. In syngeneic tumor-bearing mice, M2 TAMs express arginase-1, leading to depletion of L-arginine that is required for T cell activation [67]. These studies suggest that TAMs are mainly immunosuppressive in cancer patients as well as tumor-bearing mice.

Human TAMs secrete epidermal growth factor (EGF) to potentiate the invasiveness of ovarian cancer cells (Figure 2B) [4]. Both human and mouse TAMs upregulate MMP that degrades interstitial collagen and upregulates the synthesis and assembly of collagens to remodel TME that favors invasion of tumor cells [7,68,69]. In RCC-bearing mice, TAMs produce pro-angiogenic factors, such as VEGF, and blocking of VEGFR2 abrogates angiogenesis, indicating that TAMs exert a VEGF-dependent pro-angiogenic effect [31]. In mice with breast cancer, TAMs are shown to recruit endothelial cells, fibroblasts, and pericytes for vasculogenesis in TME [8]. Accumulating data suggest that both human and mouse TAMs exert mainly pro-tumor effects.

## 4. Dendritic Cells (DCs)

**Figure 3:**
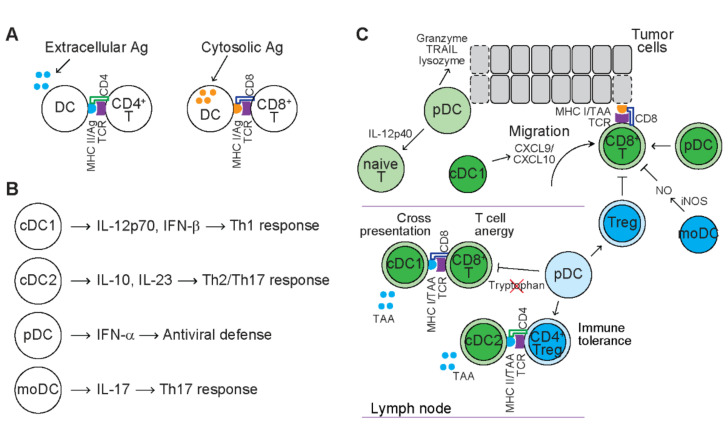
The function of DCs in tumors. (**A**) Antigen presentation of DCs. Extracellular antigens (Ag) are presented by class II major histocompatibility complex (MHC II) and recognized by CD4^+^ T cells. Cytosolic antigens are presented by class I MHC (MHC I) and recognized by CD8^+^ T cells. TCR: T-cell receptor. (**B**) Subpopulations of human DCs in physiological processes. cDC: conventional DCs; pDC: plasmacytoid DCs; moDC: monocyte-derived DCs; IL-12p70: interleukin 12p70; IFN-α: interferon beta; IL-10: interleukin 10; IL-23: interleukin 23; IFN-α: interferon alpha; IL-17: interleukin 17. (**C**) The function of DC subsets in tumors. Dark green circles represent mouse immune cells with anti-tumor function. Dark blue circles represent mouse immune cells with pro-tumor function. Light green circles represent human immune cells with anti-tumor function. Light blue circles represent human immune cells with pro-tumor function. Cells with dashed lines represent dead cells. In draining lymph node, cDC1 present extracellular tumor associated antigens (TAA) to CD8^+^ T cells through cross presentation. In tumor microenvironment (TME), cDC1 secrete C-X-C chemokine ligand 9/10 (CXCL9/10) to recruit activated CD8^+^ T cells that recognize the same TAA on tumor cells and mediate cytotoxicity of tumor cells. cDC2 present extracellular TAA to CD4^+^ regulatory T cells (Treg), leading to immune tolerance. pDCs induce pro-tumor immunity through sustaining expansion of Tregs but inhibiting proliferation of T cells through depleting tryptophan. pDCs also induce anti-tumor immunity through priming T cells and direct killing of tumor cells. moDCs suppress T cell proliferation through producing NO.

DCs are the most potent antigen presenting cells (APCs), bridging innate immunity and adaptive immunity. DCs are ontogenetically heterogeneous. Some subsets of DCs, such as plasmacytoid DCs (pDCs), conventional DCs (cDCs) and monocyte-derived DCs (moDCs), are originated from common myeloid progenitors (CMPs) expressing fms-like tyrosine kinase 3 (Flt3), macrophage-DC progenitor (MDPs) or circulating monocyes, known as the myeloid origin [70]. Some subsets of DCs, such as pDCs and cDCs, are derived from Flt3^+^ commom lymphoid progenitors (CLPs) or T cell precursors, known as the lymphoid origin [70]. DCs are phenotypically and functionally heterogeneous under physiological conditions. In response to microbial infection, extracellular microbial proteins are generally phagocytosed or endocytosed by mature DCs and presented through class II major histocompatibility complex (MHC) molecules to CD4^+^ T cells. In contrast, cytosolic microbial proteins are generally presented through class I MHC molecules to CD8^+^ T cells (Figure 3A). DCs infiltrated in TME include different subsets of DCs at different developmental stages [71]. The presence of mature DCs in TME is usually associated with favorable prognosis in cancer patients as well as tumor-bearing mice [72,73,74]. These tumor-associated DCs exert immunostimulatory or immunosuppressive effects depending on the subset of DCs and stages of tumors (Figure 3) [71,75].

### 4.1. Conventional DCs and Their Function in Tumors

Conventional DCs (cDCs), also known as classical DCs, consist of two phenotypically and functionally distinct subpopulations. Human cDC1 express CD11c, MHC-II, BDCA3, CD141, XCR1, CLEC9A, and DNGR1, while mouse cDC1 express CD11c, MHC-II, BDCA3, CD141, CD8a, and CD103 [76]. Human cDC1 express toll-like receptors (TLRs) and secrete pro-inflammatory cytokines, including interleukin 12p70 (IL-12p70) and interferon beta (IFN-α), in response to infection to induce Th1 response [77]. cDC1 are observed within tumors and their presence is associated with favorable clinical outcome [72,78,79]. In tumor-bearing mice, cDC1 in TME secrete chemokines, such as C-X-C motif chemokine ligand 9 (CXCL9) and CXCL10 to recruit effector T cells to TME [80]. Through a cross-presentation mechanism, both human and mouse cDC1 are capable of capturing extracellular proteins from tumor materials, processing tumor associated antigens (TAA) through class I MHC molecules and presenting to CD8^+^ T cells that mediate cytotoxicity of tumor cells [72,81,82,83]. In an immunocompetent melanoma mouse model, CD103^+^CD141^+^ cDC1 that carry TAA migrate to draining lymph nodes (dLN) in a C-C motif chemokine receptor 7 (CCR7)-dependent manner and present TAA to naïve CD8^+^ T cells residing in dLN, leading to T cell priming [82]. Primed CD8^+^ T cells then activate, proliferate and differentiate into cytotoxic T cells and infiltrate TME [82]. These cytotoxic T cells recognize the same TAA presented by tumor cells and mediate cytotoxicity of tumor cells, leading to prolonged survival of tumor-bearing mice [82]. With human tumor samples and mouse tumor models, CD103^+^ cDC1 exhibit strong capacity to stimulate cytotoxic T cells, which is dependent on transcription factors, such as interferon regulatory factor 8 (IRF8), zinc finger and BTB domain containing 46 (Zbtb46) and basic leucine zipper ATF-like transcription factor (Batf3) [72]. These studies suggest that cDC1 stimulate anti-tumor immune response. Consistently, cDC1 specific gene signatures are used as positive prognostic markers in cancer patients [72,78,79]. 

cDC2 are more abundant and express CD11c, MHC-II, BDCA1, CD172a (signal regulatory protein alpha, SIRPα), CD115 (CSF-1R), and CD11b [84]. Human cDC2 produce various cytokines, such as IL-10 and interleukin 23 (IL-23), and present antigens to CD4^+^ helper T cells (Th), leading to activation of effector T cells, including Th2 cells and Th17 cells [85]. cDC2 identified in both human and mouse tumors are capable of capturing extracellular TAA proteins, processing through class II MHC molecules and presenting to CD4^+^ T cells [86]. A two-step priming model is proposed in that cDC1 prime CD8^+^ T cells while cDC2 prime CD4^+^ T cells in distinct regions of dLN [87]. In secondary immune response, the same cDC1 activates both CD4^+^ T cells and CD8^+^ T cells [88]. CD4^+^ T cells produce cytokines and provide costimulatory signals to promote clonal expansion of CD8^+^ T cells and their differentiation into cytotoxic T cells and memory T cells [88]. Despite the observation that migratory cDC2 stimulate the priming, activaton and expansion of CD4^+^ T cells, the effector T cells are mainly Tregs that suppress the function of cDC2 and result in immune tolerance [86]. Depletion of Tregs restores the function of cDC2, leading to differentiaton of primed CD4^+^ T cells to effector T cells that mediate anti-tumor immune response [86]. In the absence of Tregs, the amount of cDC2 correlates with the number of CD4^+^ T cells and responsiveness to checkpoint inhibitors [86]. In human and mouse lung cancer, expression of cDC2 gene signature is associated with positive prognosis [89]. Therefore, both cDC1 and cDC2 favor anti-tumor immunity.

### 4.2. Plasmacytoid DCs and Their Function in Tumors

DCs that share morphology with antibody-secreting plasma cells are known as pDCs. Human pDCs are CD123^+^CD303^+^CD304^+^CD11c^−^, while mouse pDCs are CD11c^low^B220^+^CD317^+^Siglec-H^+^ (sialic acid-binding immunoglobulin-like lectin H) [76]. In response to viral infection, pDCs recognize viral nucleic acids via TLRs and secrete type I interferon IFN-α and play a key role in antiviral defense [90]. pDCs are observed in TME of cancer patients and are associated with early relapse and unfavorable clinical outcome [11,91,92,93,94]. In aggressive triple-negative breast tumor samples, pDCs colocalize with Tregs and exhibit partially activated phenotype with impaired IFN-α production upon engagement of TLRs [93]. Reduced production of IFN-α by tumor-infiltrating pDCs sustains the expansion of FoxP3^+^ Tregs in vivo, contributing to immune tolerance and progression of breast cancer [93]. Human head and neck squamouse cell carcinoma (HNSCC) cells dampen the function of pDCs through downregulating TLR expression, leading to reduced production of IFN-α and impaired T cell-mediated anti-tumor immune response [94]. pDCs are also found in tumor dLN in melanoma patients [92]. These dLN pDCs express indoleamine 2,3-dioxygenase that deplete tryptophan (amino acids critical for T cell proliferation), leading to T cell anergy and immune tolerance [9]. This evidence suggests that pDCs induce an immunosuppressive immune response.

In addition to immunosuppressive effects, some studies reveal that pDCs induce immunostimulatory response. CD123^+^ pDCs are located in peritumoral areas of primary melanomas in close contact with CD8^+^ T cells [95]. Functional assays reveal that both human and mouse pDCs prime CD8^+^ T cells, resulting in their activation and differentiaton to cytolytic and IFN-γ-producing effector T cells and regression of tumors in vivo [95,96]. Human CD2^high^ pDCs infiltrated in TME express high levels of granzyme B, TRAIL and lysozyme, which limit proliferation of tumor cells and mediate contact-dependent killing of tumor cells [97]. In addition, CD2^high^ pDCs are also efficient at secreting IL-12p40, which primes naïve T cells and results in T cell expansion and immune response [97]. Therefore, DCs exert both pro-tumor and anti-tumor effects during tumor development.

### 4.3. Monocyte-Derived DCs and Their Function in Tumors

In response to infection, circulating monocytes enter tissue and differentiate into DCs [98]. These DCs are known as moDCs or inflammatory DC (inf-DC), and express CD1a, BDCA1, CD11c, MHC II, and CD64 in humans and CD11b, CD11c, F4/80, CD64, FceRIα, Ly6C, and MHC II in mice [76]. MoDCs are absent under homeostatic conditions, but observed in inflammatory cues, such as inflammaotory fluids, and secrete interleukin 17 (IL-17) to induce differentiation of naïve CD4^+^ T cells into Th17 cells [99]. MoDC are also observed in TME of certain cancers, such as lung cancer and breast cancer mouse models as well as human cancers [71]. With a lung carcinoma mouse model, CD64^hi^CD11b^+^ MoDCs are observed in TME [71]. Adoptive transfer experiments reveal that tumor-infiltrating MoDCs are derived from Ly6C^+^ bone marrow monocytes [71]. Deletion of CCR2 results in the lack of MoDCs in TME, indicating that migration of circulating Ly6C^+^ monocytes to TME is dependent on CCR2 [71]. Despite high efficiency in uptaking and processing antigens, MoDCs exhibit low efficiency on activating naïve T cells [71]. Instead, MoDCs are efficient at expressing TNF-α and inducible nitric oxide synthase (iNOS) [71]. MoDCs therefore phenotypically recapitulate the TNF-α-iNOS-producing DCs (Tip-DCs) and iNOS-mediated production of NO suppresses T cell proliferation [71], indicating that MoDCs induce immunosuppressive response. With a lymphoma mouse model, the anti-tumor effect mediated by adoptively transferred CD8^+^ T cells is dependent on nitric oxide synthase 2 (NOS2) expressed by Tip-DCs [100]. Tip-DCs improve the anti-tumor effect of CD8^+^ T cells through mediating NO-dependent killing of lymphoma cells, indicating that Tip-DCs exert immunostimulatory effect. Accordingly, gene signatures of Tip-DCs are positively associated with genes expressed in active CD8^+^ cytotoxic T cells and survival of colorectal cancer patients [100]. The discrepancy between these two studies may be due to response of T cells and tumor cells to NO. Therefore, the function of MoDCs in tumors needs further study.

## 5. Granulocytes

### 5.1. Cancer-Related Circulating Neutrophils

**Figure 4:**
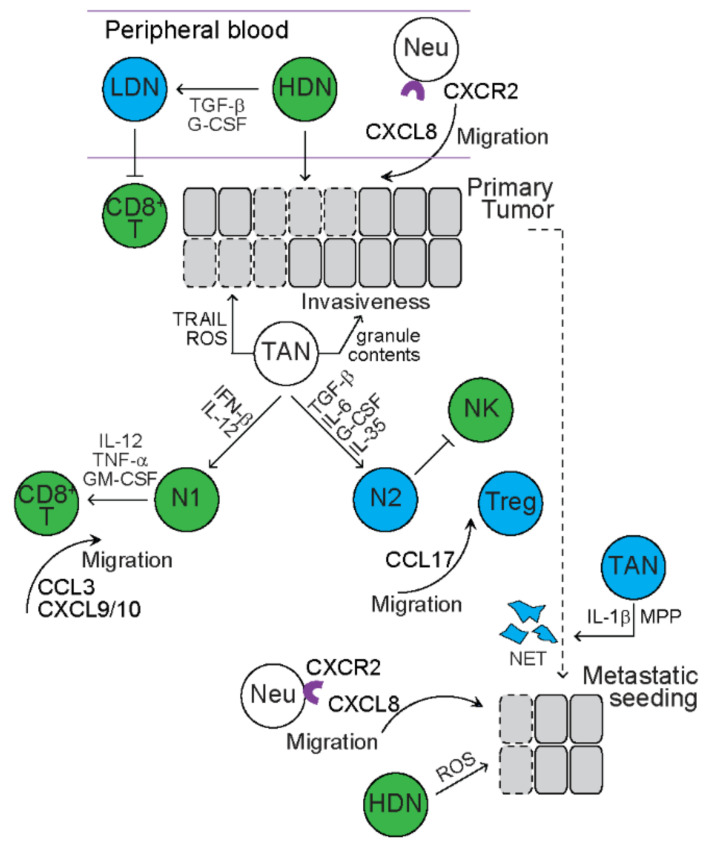
The function of neutrophils in tumors. Dark green circles represent mouse immune cells with anti-tumor function. Dark blue circles represent mouse immune cells with pro-tumor function. White circles represent mouse immune cells with both anti-tumor and pro-tumor function. Cells with dashed lines represent dead cells. Transforming growth factor beta (TGF-β) and granulocyte colony stimulating factor (G-CSF) induce transition of high-density neutrophils (HDN) to low density neutrophils (LDN) with tumor progression. HDN induce cytotoxicity of primary tumor cells and after metastasis, while LDN limit the proliferation of CD8^+^ T cells. C-X-C motif chemokine receptor 2 (CXCR2)/C-X-C chemokine ligand 8 (CXCL8) axis mediates recruitment of neutrophils (Neu) to primary and metastatic tumors. Tumor-associated neutrophils (TAN) promote invasiveness of cancer cells through releasing granule contents, promote dissemination of cancer cells by secreting interleukin 1 beta (IL-1β) and matrix metalloproteinases (MPP). Neutrophil extracellular traps (NET) promote metastasis through catching cancer cells. TAN mediate cytotoxicity of tumor cells through producing tumor necrosis factor-related apoptosis-inducing ligand (TRAIL) and reactive oxygen species (ROS). TANs are polarized to anti-tumor N1 TANs (N1) or pro-tumor N2 TANs (N2) depending on TME cues. N1 TANs inhibit natural killer (NK) cell function while recruit regulatory T cells (Treg) to TME. N1 TAN secrete chemokines to recruit CD8^+^ T cells to TME and secrete cytokines to activate CD8^+^ T cells. IFN-β: interferon beta; IL-12: interleukin 12; IL-6: interleukin 6; IL-35: interleukin 35; TNF-α: tumor necrosis factor alpha; GM-CSF: granulocyte-macrophage colony stimulating factor; CCL3: C-C motif chemokine ligand 3; CXCL9/10: C-X-C chemokine ligand 9/10; CCL17: C-C motif chemokine ligand 17.

Neutrophils are polymorphonuclear phagocytes that circulate peripheral blood and function as innate immunity against microbial pathogens. In patients with cancer, especially at advanced stages and after metastasis, the number of circulating neutrophils increases and high neutrophil-to-lymphocyte ratio (NLR) is associated with aggressive outcome [101,102,103]. Similarly, in tumor-bearing mice, the number of neutrophils also increases in peripheral blood and is also associated with aggressive outcome and metastatic potential [104,105]. These cancer-related circulating neutrophils comprise functionally heterogenous subsets (Figure 4). With Ficoll-Hypaque density gradient centrifugation, neutrophils are found in the high-density (HD) fraction (high-density neutrophils, HDNs), while mononuclear cells are found in the low-density (LD) fraction [105]. HDNs are a homogeneous group of mature, segmented neutrophils, expressing CD66b, CD11b, CD15, CD16, and CD10 in humans and CD11b, Gr1, and Ly6G in mice [105,106]. Neutrophils are also found in the LD fraction (low-density neutrophils, LDNs) [105]. LDNs are a heterogenous group of cells with two major subpopulations based on developmental stages—i.e., mature neutrophils that are derived from HDNs and immature MDSCs [105]. In tumor-free mice, over 95% of circulating neutrophils are HDNs [105]. In tumor-bearing mice, such as breast cancer, mesothelioma and lung cancer, the number of circulating neutrophils, especially LDNs, increases with tumor progression [105]. Increased proportion of LDNs is partly due to TGF-β-dependent sponteneous transition from HDNs, especially in mice bearing tumors in late-stages [105]. HDNs exhibit high levels of phagocytosis and mediate cytotoxicity of tumor cells in vitro [105]. In initial tumor growth, adoptive transfer of HDNs dramatically retards tumor growth in vivo [105]. In both spontaneous and experimental metastatic mouse models, CD11b^+^MMP^+^ HDNs (also refered as tumor entrained neutrophils, TENs) accumulate in the pre-metastatic lung, and inhibit seeding of tumor cells in the pre-metastatic area through mediating direct cytotoxicity of tumor cells through releasing ROS [107]. In contrast, LDNs exhibit reduced ROS production, no cytotoxicity towards tumor cells and no significant effect on initial tumor growth [105]. Mechanistic studies reveal that LDNs induce a supportive TME through downregulating expression of pro-inflammatory cytokines and limiting proliferation of CD8^+^ T cells [105]. With a breast cancer mouse model, granulocyte colony-stimulating factor (G-CSF) derived from tumor cells is shown to reprogram neutrophils toward immunosuppressive LDNs [108]. These studies suggest that HDNs exert anti-tumor effects, while LDNs exert pro-tumor effects.

### 5.2. Tumor-Associated Neutrophils (TANs)

In addition to cancer-related circulating neutrophils, TANs also play a critical role in tumor development and metastasis (Figure 4). Most C-X-C motif chemokine receptor 2 (CXCR2)^+^ leukocytes in peripheral blood are neutrophils. In spontaeous tumor mouse models, inhibition of CXCR2 reduces the infiltration of Ly6G^+^ neutrophils and tumor formation [109]. In a Kras^LAI^-driven lung adenocarcinoma mouse model, CXCR2 ligand, C-X-C motif chemokine ligand 8 (CXCL8), is a transcriptional target of Ras and induces progression of premalignant lesion that is associated with infiltration of neutrophils [110]. These studies suggest that CXCR2 mediates recruitment of neutrophils to primary tumor sites. In a mouse model recapitulating human pancreatic ductal adenocarcinoma, deletion or inhibition of CXCR2 abrogates metastasis [111]. In a breast cancer mouse model, metastasis of tumor cells to lung is abolished by inhibiting CXCR2, which is associated with reduced recruitment of neutrophils [112]. These studies suggest that CXCR2 also mediates recruitment of neutrophils to a premetastatic site.

Multiple studies demonstrate that TANs in TME promote proliferation, extravasation, and migration of tumor cells (Figure 4). With esophageal cancer cell lines, TANs are shown to release granule contents, such as elastase, to promote proliferation and invasion of cancer cells [113]. TANs are shown to promote dissemination of cancer cells by secreting IL-1β and MPPs to facilitate extravasation of tumor cells to premetastatic niches [114]. Cools-Lartigue et al. reveal that neutrophil extracellular traps (NET) catch circulating lung carcinoma cells, which activates TLR pathway to promote migration, adhesion, invasion and metastasis of tumor cells [115,116]. In addition to pro-tumor effects, TANs are also shown to mediate cytotoxicity of tumor cells through producing ROS and TRAIL [117,118]. 

The opposing effects of neutrophils on tumor development and metastasis implicate functional plasticity of neutrophil subsets (Figure 4). Neutrophils with anti-tumor effect are termed “N1” TANs, while neutrophils with pro-tumor effect are termed “N2” TANs [119]. Studies propose that TME impacts on the balance of N1 and N2 subpopulations through secreting cytokines that reprogram neutrophil differentiation. For example, TGF-β, IL-6, G-CSF and interleukin 35 (IL-35) are shown to induce pro-tumor polarization of TANs [119,120,121]. IFN-β and IL-12 are shown to induce anti-tumor polarization of TANs [122,123]. Studies have also shown that TANs interact with lymphocytes in TME and regulate their functions. Natural killer (NK) cells function as immunosurveillance to clear tumor cells from dissemination during metastasis. In 4T1-bearing mice, N2 TANs inhibit NK cell-mediated clearance of tumor cells, thus promoting tumor metastasis [114]. N2 TANs secrete CCL17 to recruit Tregs into TME, thus promoting tumor growth [124]. In contrast, N1 TANs produce chemokines, such as C-C motif chemokine ligand 3 (CCL3, CXCL9, CXCL10), to recruit CD8^+^ T cells to TME and secrete cytokines (e.g., IL-12, TNF-α, and GM-CSF) to activatethe cytotoxicity of CD8^+^ T cells [119], thus providing anti-tumor effect.

### 5.3. Other Types of Granulocytes

In addition to neutrophils (neutrophilic granulocytes), other types of granulocytes, such as eosinophilic granulocytes (eosinophils) and basophilic granulocytes (basophils), also impact on tumors. Eosinophils are known for their roles in defense againt helminth infections and allergic diseases. With co-culture system, both human and mouse eosinophils exhibit direct cytotoxicity to various cancer cells, such as mastocytoma, melanoma, and colon carcinoma [125,126,127]. Eosinophils also exhibit indirect cytotoxicity to cancer cells through secreting pro-inflammatory cytokines, such as TNF-α [128]. In mice bearing hepatocellular carcinoma, interleukin 5 (IL-5) activates eosinophils, leading to suppression of tumor growth [128]. In mice bearing melanoma, administration of interleukin 33 (IL-33) delays tumor growth and prevents pulmonary metastasis through recruiting and activating eosinophils [126]. Mechanistic studies reveal that IL-33 stimulates contact-dependent degranulation of eosinophils and resultant killing of tumor cells [129]. In mice bearing melanoma, colorectal carcinoma, thymoma or lung carcinoma, transfer of eosinophils enhances recruitment of CD8^+^ T cells to suppress tumor growth that is associated with increased expression of pro-inflammatory cytokines, decreased expression of pro-angiogenic factors, and M1-like polarization of TAMs [130]. These studies indicate anti-tumor activity of eosinophils. Intriguingly, infiltration of eosinophils is observed in patients with cervical cancer [131]. Mechanistic studies reveal that thymic stromal lymphopoietin (TSLP) activates eosinophils that promote proliferation and survival of cervical cancer cells through upregulating Ki-67, proliferating cell nuclear antigen (PCNA) and BCL-2 and downregulating Fas and Fas ligand (FasL) [131]. In addition, TSLP stimulates eosinophils to produce VEGF and IL-8 that promotes angiogenesis in vitro [132]. Eosinophils also induce M2-like polarization of TAMs through secreting IL-13 [133]. These studies indicate a pro-tumor activity of eosinophils. In clinic, infiltration of eosinophils in TME correlates to favorable prognosis in patients with colonic carcinomas, oral squamous cell carcinomas, esophageal squamouse cell carcinoma, and nasopharyngeal carcinoma [134,135,136,137]. However, accumulation of eosinophils in tumors correlates with shorter survival time in patients with Hodgkin’s disease, a group of B cell malignancies [138]. Discrepancies of eosinophil-mediated immunity in tumors may be due to plasticity of eosinophils or heterogeneity of tumors, which needs further study.

Basophils are very rare in peripheral blood and are known to defend against parasites. Accumulation of basophils in bone marrow of patients with myelodysplastic syndrome (MDS), a pre-leukemia condition, is an independent prognostic factor for evolution to AML [139,140]. Growth of basophils from patients with chronic myeloid leukemia (CML) is associated with unfavorable prognosis and transformation to AML [141]. Mechanistic studies reveal that basophils secrete hepatocyte growth factor (HGF), leading to expansion of CML cells [142]. These studies indicate that basophils may be involved in disease evolution to high-risk hematologic malignancies. In contrast, colorectal cancer patients with low basophil count in peripheral blood exhibit shorter disease free survival time [143], indicating the anti-tumor potential of basophils. In addition, melanoma patients with high levels of basophils exhibit favorable overall survival after immunotherapy [144]. Breast cancer-bearing mice with low frequency of basophils in peripheral blood exhibit increased lung metastases [145]. Given the difficulty of deleting or depleting basophils in vivo, the function of basophils in tumors is still unclear and needs more investigation.

## 6. Myeloid-Derived Suppressor Cells (MDSCs)

### 6.1. Function of MDSCs in Tumors

**Figure 5:**
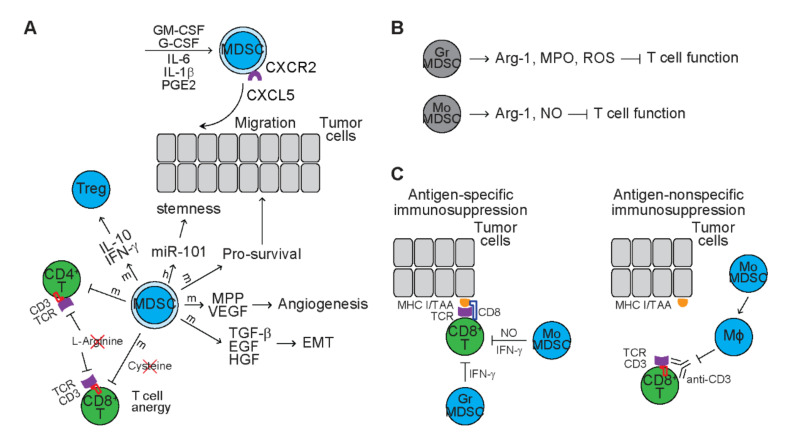
The function of MDSCs and MDSC subsets in tumors. (**A**) The function of MDSCs in tumors. Dark green circles represent mouse immune cells with anti-tumor function. Dark blue circles represent mouse immune cells with pro-tumor function. Light blue circles represent human immune cells with pro-tumor function. Various cytokines stimulate myelopoiesis, generating myeloid- derived suppressor cells (MDSC). The C-X-C chemokine ligand 5 (CXCL5)/ C-X-C motif chemokine receptor 2 (CXCR2) axis induces recruitment of circulating MDSC to tumor microenvironment (TME). MDSC enhance stemness and survival cancer cells. MDSC also stimulate angiogenesis and induce epithelial–mesenchymal transition (EMT). MDSC suppress proliferation and activation of T cells through depleting L-arginine and cysteine, downregulating CD3 and disrupting T-cell receptor (TCR) function. MDSC also induce regulatory T cells (Treg) through secreting interleukin 10 (IL-10) and interferon gamma (IFN-γ). GM-CSF: granulocyte-macrophage colony stimulating factor; G-CSF: granulocyte colony stimulating factor; IL-6: interleukin 6; IL-1β: interleukin 1 beta; PGE2: prostaglandin E2; MMP: matrix metalloproteinases; VEGF: vascular endothelial growth factor; TGF-β: transforming growth factor beta; EGF: epidermal growth factor; HGF: hepatocyte growth factor; m: mouse; h: human. (**B**) MDSC subsets in tumor-bearing mice. Granulocytic myeloid-derived suppressor cells (GrMDSC) suppress T cell function through producing reactive oxygen species (ROS). Monocytic myeloid-derived suppressor cells (MoMDSC) suppress T cell function through producing nitric oxide (NO). Arg-1: arginase-1; MPO: myeloperoxidase. **(C)** Immunosuppression mediated by MDSC subsets. GrMDSC mediate IFN-γ-dependent antigen-specific immunosuppression. MoMDSCs mediate antigen-specific immunosuppression that is required for IFN-γ and NO (left panel). MoMDSC-derived macrophages (Mϕ) mediate antigen-nonspecific immunosuppression. MHC I: class I major histocompatibility complex; TAA: tumor-associated antigen.

MDSCs are a heterogenous group of myeloid progenitors and immature myeloid cells at distinct developmental stages that circulate the blood vessels [146]. In response to infection, MDSCs rapidly expand and differentiate into granulocytes, monocytes, macrophages, and DCs and play an essential role in the regulation of immune responses and tissue repair [146]. Human MDSCs express CD14^−^, CD11b^+^, CD33^+^, and class II MHC^−^, while mouse MDSCs express CD11b^+^, Gr-1^+^ and the marker for immature myeloid cells CD31^+^ [147,148]. In cancer patients, the number of circulating MDSCs increases and the infiltration of MDSCs in TME is associated with poor prognosis [149,150,151]. Tumor cells or TAMCs produce GM-CSF, G-CSF, and IL-6 that drive CCAAT/enhancer binding protein beta (C/EBPβ)-dependent myelopoiesis from myeloid progenitor cells in human and mouse bone marrow, generating MDSCs (Figure 5A) [152]. Mice bearing tumors that secrete IL-1β also induce myelopoiesis, leading to increased number of MDSCs in peripheral blood and spleens [153]. Deletion or blocking of prostaglandin E2 (PGE2) reduces the number of MDSCs via blocking differentiation of MDSCs from immature hematopoietic cells, indicating that PGE2 induces differentiation of MDSCs [154]. In a spontaneous melanoma mouse model, C-X-C motif chemokine ligand 5 (CXCL5) induces recruitment of CXCR2^+^ MDSCs to the primary tumor [155].

MDSCs exert various pro-tumor effects during tumor progression and metastasis (Figure 5A). Coculture of MDSCs with primary ovarian cancer cells increases the aldehyde dehydrogenase (ALDH)^+^ cancer stem cells, promotes tumor sphere formation in vitro, and increases tumor incidences and metastatic foci in vivo in xengraft models, indicating that MDSCs enhance cancer stemness [151]. Mechanistic studies reveal that MDSCs upregulates the expression of microRNA101 that represses transcription factor C-terminal binding protein-2 (CtBP2), leading to upregulation of stem cell core genes, such as *NANOG*, *OCT4/3*, and *SOX2* [151]. In tumor-bearing mice, transfer of MDSCs reduces apoptosis and necrosis of tumor cells, indicating that MDSCs provide pro-survival signals for tumor cells [156]. MDSCs accumulated in TME produce MMP9 to promote tumor growth and tumor vasculature [156]. These tumor-associated MDSCs acquire endothelial cell properties and incorporate into tumor vasculature [156]. MDSCs also secrete VEGF in a Stat3-dependent manner, which initiates angiogenesis through inducing endothelial cell migration and tube formation [157]. In melanoma-bearing mice, MDSC accumulated in TME produce TGF-β, EGF, and HGF to induce epithelial-mesenchymal transition (EMT), facilitating cancer cell dissemination [155].

The main feature of MDSCs is to suppress the function of immune cells with a focus on T cells (Figure 5A). Tumor-associated lineage^−^CD45^+^CD33^+^ MDSCs from high-grade ovarian serous cancer patients suppress proliferation of T cells and inhibit their function through downregulating the expression of IL-2, INF-γ, and granzyme B, resulting in increased tumor volume [151]. MDSCs also reduce the number of antigen-specific CD8^+^ T cells and inhibit the cytoxoticity of CD8^+^ T cells [152]. Mechanistic studies reveal that tumor-associated MDSCs in mice bearing lung carcinoma express high levels of arginase-1 and cationic amino acid transporter 2B that deplete extracellular L-Arginine, leading to downregulated expression of CD3zeta and reduced proliferation of antigen-specific T cells [158]. Activation and proliferation of T cells requires importing of cysteine from APCs, such as macrophages and DCs [159]. MDSCs limit the cysteine supply of T cells by sequestering cysteine from extracellular space, leading to suppression of T cell expansion [159]. In tumor-bearing mice, MDSCs also disrupt the binding of T cell receptor (TCR) to MHC-antigen complex through nitration of TCR/CD8, leading to T cell anergy [160,161]. In colon carcinoma mouse model, MDSCs inhibit proliferation of CD4^+^ T cells driven by antigens, but induce the development of anergic and suppressive Tregs that is dependent on IL-10 and IFN-γ [162].

### 6.2. Subpopulations of Tumor-Associated MDSCs

MDSCs consist of two main subpopulations (Figure 5B). Granulocytic MDSCs (GrMDSCs) are phenotypically and morphologically similar to neutrophils and express CD11b^+^CD33^+^CD14^−^CD15^+^ in humans and CD11b^+^Ly6G^+^Ly6C^low^ in mice [163,164]. In tumor-free mice, neutrophils exhibit high levels of phagocytosis and express high levels of lysosomal enzymes and TNF-α [165]. In tumor-bearing mice, GrMDSCs exhibit lower levels of phagocytosis but higher levels of activation or production of arginase-1, myeloperoxidase (MPO) and ROS that suppress T cell function [163,164,165]. For example, ROS reduce expression of TCR zeta chain and decrease cytokine production, leading to impaired T cell activation [166]. Monocytic MDSCs (MoMDSCs) are phenotypically and morphologically similar to monocytes and express CD14^+^HLA-DR^low^ in humans and CD11b^+^Ly6G^−^Ly6C^high^ in mice [148,163,164]. Compared to monocytes, MoMDSCs exhibit high levels of activation or production of arginase-1 and NO and the ability to suppress T cell function [163,164]. NO impairs interleukin 2 (IL-2) downstream signaling pathways, including JAK3/STAT5, ERK, and AKT, leading to suppression of T cell activation and proliferation driven by antigens or mitogens [167].

Inoculation of T-cell lymphoma cells induces expansion of GrMDSCs and MoMDSCs in spleens of recipient mice [163]. Both GrMDSCs and MoMDSCs suppress proliferation of CD8^+^ T cells driven by ovalbumin protein, indicating antigen-specific immunosuppression (Figure 5C) [163]. Blocking of IFN-γ completely reverses the suppressive effect of GrMDSCs on T cells but only partially reverses the suppressive effect of MoMDSCs on T cells, indicating that GrMDSCs mediate immunosuppression in an IFN-γ-dependent manner [163]. Inhibition of iNOS partly reverses the suppressive effect of MoMDSCs on T cells but shows no alteration on the suppressive effect of GrMDSCs on T cells, indicating that MoMDSCs supress T cell proliferation via the IFN-γ and iNOS/NO pathways [163]. In addition, macrophages differentiated from MoMDSCs suppress proliferation of CD8^+^ T cells driven by anti-CD3 antibodies, indicating antigen-nonspecific immunosuppression [163]. Inhibition of iNOS completely reverses while blocking of IFN-γ partially reverses suppressive effect of MoMDSC-derived macrophages on polyclonal T cell proliferation [163]. These studies suggest that both GrMDSCs and MoMDSCs contribute to the immunosuppression of T cells.

## 7. Tumor-Associated Myeloid Cells in Cancer Therapy

### 7.1. Chemotherapy

Accumulating evidence reveal that TAMCs contribute to chemoresistance [168,169,170]. For example, TAMs release putrescine that protects colorectal cancer cells from 5-fluorouracil (5-FU)-mediated apoptosis [13]. In mice bearing lung carcinoma or breast cancer, M2 TAMs accumulate around TME vasculature after chemotherapy and release VEGF that promotes angiogenesis and relapse [14]. In response to paclitaxel, the anti-microtubule chemotherapeutic agent, macrophages infiltrate TME in mice bearing mammary tumors and secrete cathepsin to protect tumor cells from chemotherapy-mediated cell death [171]. Inhibition of cathepsin enhances the response to chemotherapy in tumor-bearing mice, leading to impaired tumor growth and metastasis in the recipient [171]. 

Therapeutic approaches targeting TAMCs as monotherapy or in combination with chemotherapy is being tested in preclinical and clinical settings. Among the heterogeneous population of TAMCs, TAMs, and MDSCs are mainly pro-tumoral and immunosuppressive, and are therefore being tested in different tumors. For example, targeting the recruitment of monocytes and TAMs to TME are being vigorously tested in animal models and patients with tumors. TAMs are recruited to TME by CSF-1 and promote breast cancer development and metastasis [52]. Therefore, therapeutic approaches targeting the CSF-1/CSF-1R axis with mAb or small molecule compounds are being tested. In a mouse model bearing chemoresistant MCF-7 breast cancer cells, injection of a murinized antigen-binding fragment targeting mouse CSF-1 retards tumor growth, enhances response to chemotherapeutic agents—including cyclophosphamide, methotrexate, and 5-FU—and prolongs survival of the mice [172]. Humanized mAb (RG7155) suppressing human CSF-1R activation induces cell death of immunosuppressive M2-like macrophages in vitro [173]. In mice bearing colorectal adenocarcinoma and fibrosarcoma, treatment with chimeric mouse antibody suppressing mouse CSF-1R depletes TAMs, resulting in increased CD8/CD4 T cell ratio [173]. Treatment of RG7155 in seven patients diagnosed with diffuse-type giant cell tumor (Dt-GCT) results in partial clinical response in all patients and complete clinical response in two patients (clinicaltrials.gov identifier NCT01494688) [173]. In addition, the amount of CD68^+^CD163^+^ macrophages is reduced in tumor biopsies from RG7155-treated Dt-GCT patients, indicating reduced recruitment of TAMs to TME [173]. In mice bearing glioblastoma multiforme or patient-derived glioma, BLZ945, a brain-penetrant kinase inhibitor targeting CSF-1R depolarizes M2 phenotype of TAMs, impairs their pro-tumor function and slows tumor growth that is associated with decreased M2 gene signatures [174]. In a glioma mouse model, a tyrosine kinase inhibitor PLX3397 that targets CSF-1R, c-Kit and Flt3 blocks tumor progression through depolarizing M2 phenotype of TAMs and impairing their pro-tumor function [175]. PLX3397 is also tested in patients with melanoma (clinicaltrials.gov identifier NCT02071940, NCT02975700), prostate cancer (clinicaltrials.gov identifier NCT01499043), and glioblastoma (clinicaltrials.gov identifier NCT01349036). Other CSF-1R inhibitors, such as ARRY-382 (clinicaltrials.gov NCT01316822), BLZ945 (clinicaltrials.gov identifier NCT02829723), AMG820 (clinicaltrials.gov identifier NCT01444404), and IMC-CS4 (clinicaltrials.gov identifier NCT01346358) are also tested in patients with various solid tumors.

In addition to monotherapy, inhibitors targeting CSF-1 or CSF-1R are also tested in combination with anti-cancer therapy for safety, tolerability and efficacy. For example, PLX3397 is tested in patients with advanced solid tumors in combination with paclitaxel (clinicaltrials.gov identifier NCT01525602) [176]. PLX3397 is tested in combination with eribulin in patients with breast cancer (clinicaltrials.gov identifier NCT01596751). PLX3397 is also tested with vemurafenib in patients with BRAF-mutated melanoma (clinicaltrials.gov identifier NCT01826448). PLX3397 is tested with sirolimus in patients advanced sarcomas (clinicaltrials.gov identifier NCT02584647). RG7155 is tested with paclitaxel in patients with ovarian cancers or breast cancers (clinicaltrials.gov identifier NCT01596751). 

### 7.2. Immunotherapy

Upon binding to ligands CD80/CD86 expressed on APC and PD-L1 expressed on tumor cells, the immune checkpoint receptors, cytotoxic T-lymphocyte-associated protein 4 (CTLA-4) and PD-1, expressed on T cells induce inhibitory signals, leading to inactivation of T cells and immune evasion [177,178]. Inhibitors targeting CTLA-4 and PD-1 block these inhibitory pathways, leading to reactivation of anti-tumor immunity and clinical benefit in some cancer patients [177,178]. However, many cancer patients are resistant to immune checkpoint inhibitors [177,178]. Increasing studies suggest that TAMCs, such as MDSCs and TAMs, contribute to resistance to immune checkpoint inhibitors and therapeutic approaches targeting development, recruitment and function of MDSCs and TAMs are being tested for their ability to improve sensitivity to checkpoint inhibitors. 

In melanoma patients, circulating Lin^−^CD14^+^HLA-DR^−^ moMDSCs are increased compared to healthy donors [179]. Melanoma patients responding to ipilimumab, an mAb targeting CTLA-4, exhibit less circulating moMDSCs than nonresponders [179]. Metastatic melanoma patients who fail to respond to ipilimumab are treated with nivolumab, an mAb targeting PD-1, and the frequency of circulating moMDSCs is inversely correlated to survival time [180]. In addition to circulating MDSCs, the accumulation of MDSCs in TME limits the clinical efficacy of checkpoint inhibitors in sarcoma patients [181]. In tumor-bearing mice, MDSCs infiltrated in TME express high levels of PD-L1 that is induced by HIF-1α [182]. In sarcoma-bearing mice, sarcoma tissue induces expansion of MDSCs that express CXCR2 [181]. Deficiency or inhibition of CXCR2, the main chemokine receptor recruiting MDSCs to TME, reduces the infiltration of CD11b^+^Ly6G^high^ MDSCs to TME, leading to enhanced anti-tumor effects upon treatment with checkpoint inhibitor [181]. In pancreatic cancer patients, the CXCR2 signaling is also overactivated [111]. In a pancreatic cancer mouse model, deletion or inhibition of CXCR2 enhances infiltration of T cells and extends survival time of mice that are treated with checkpoint inhibitors [111]. SX-682, a pharmacological inhibitor on CXCR1/2, inhibits infiltration of MDSCs to TME in oral and lung carcinoma mouse model, and enhances the effect of checkpoint inhibitors as well as T cell therapy [183]. Inhibition of CXCR2 is also tested in patients with pancreatic cancers (clinicaltrials.gov identifier NCT00851955) and metastatic melanoma (clinicaltrials.gov identifier NCT01740557). SC-682, the allosteric inhibitor of CXCR1/2, is tested in patients with myelodysplastic syndromes (clinicaltrials.gov identifier NCT04245397). SC-682 is also tested in combination with nivolumab in patients with metastatic pancreatic ductal adenocarcinoma (clinicaltrials.gov identifier NCT04477343). In addition, SC-682 is tested in combination with pembrolizumab, an mAb targeting PD-1, in patients with metastatic melanoma (clinicaltrials.gov identifier NCT03161431). 

In addition to targeting MDSCs, targeting recruitment of TAMs is also tested through inhibiting the CSF-1/CSF-1R axis in combination with checkpoint inhibitors. IMC-CS4 is tested in combination with PD-L1 inhibitor (durvalumab) or CTLA4 inhibitor (tremelimumab) in patients with solid tumors (clinicaltrials.gov identifier NCT02718911) [184]. PLX3397 is tested in combination with pembrolizumab in patients with various tumors (clinicaltrials.gov identifier NCT02452424). ARRY-382 is tested in combination with pembrolizumab in patients with solid tumors (clinicaltrials.gov identifier NCT02880371). BLZ945 is tested in combination with PDR001, an mAb targeting PD-1, in patients with solid tumors (clinicaltrials.gov identifier NCT02829723). RG7155 is tested in combination with atezolizumab, an mAb targeting PD-L1, in patients with various tumors (clinicaltrials.gov identifier NCT02323191). AMG820 is tested with pembrolizumab in patients with solid tumors (clinicaltrials.gov identifier NCT02713529).

## 8. Conclusions

Accumulating studies reveal that TAMCs play a critical role in tumor development, metastasis, immunomodulation, tumor vasculature formation, TME remodeling, and response to cancer therapy. Modulating the development, maturation and function of these myeloid cells merits the discovery of novel therapeutic strategies. However, these myeloid cells perform overlapping or opposing functions due to the complexity and plasticity of various interchangeable subpopulations. In addition, the molecular mechanism governing the behavior of TAMCs is largely unclear. Future studies will focus on further clarifying the function of each subpopulation of the myeloid cells in different cancers and identifying the molecular mechanism related to their pro-tumor and anti-tumor activities. This will help us understand the complexity of these myeloid cells and design novel targeted therapies. 

## Data Availability

Not applicable.
